# High-throughput determination of dry mass of single bacterial cells by ultrathin membrane resonators

**DOI:** 10.1038/s42003-022-04147-5

**Published:** 2022-11-11

**Authors:** Adrián Sanz-Jiménez, Oscar Malvar, Jose J. Ruz, Sergio García-López, Priscila M. Kosaka, Eduardo Gil-Santos, Álvaro Cano, Dimitris Papanastasiou, Diamantis Kounadis, Jesús Mingorance, Álvaro San Paulo, Montserrat Calleja, Javier Tamayo

**Affiliations:** 1 Instituto de Micro y Nanotecnología, IMN-CNM, CSIC (CEI UAM+CSIC), 28760 Tres Cantos, Madrid Spain; 2 Fasmatech Science & Technology, Lefkippos TESPA, Demokritos NCSR, Patriarchou Gregoriou & Neapoleos, 15341 Athens, Greece; 3 Hospital Universitario La Paz, IdiPAZ, 28046 Madrid, Spain

**Keywords:** Mass spectrometry, Biophysics, Bacteria

## Abstract

How bacteria are able to maintain their size remains an open question. Techniques that can measure the biomass (dry mass) of single cells with high precision and high-throughput are demanded to elucidate this question. Here, we present a technological approach that combines the transport, guiding and focusing of individual bacteria from solution to the surface of an ultrathin silicon nitride membrane resonator in vacuum. The resonance frequencies of the membrane undergo abrupt variations at the instants where single cells land on the membrane surface. The resonator design displays a quasi-symmetric rectangular shape with an extraordinary capture area of 0.14 mm^2^, while maintaining a high mass resolution of 0.7 fg (1 fg = 10^−15 ^g) to precisely resolve the dry mass of single cells. The small rectangularity of the membrane provides unprecedented frequency density of vibration modes that enables to retrieve the mass of individual cells with high accuracy by specially developed inverse problem theory. We apply this approach for profiling the dry mass distribution in *Staphylococcus epidermidis* and *Escherichia coli* cells. The technique allows the determination of the dry mass of single bacterial cells with an accuracy of about 1% at an unparalleled throughput of 20 cells/min. Finally, we revisit Koch & Schaechter model developed during 60 s to assess the intrinsic sources of stochasticity that originate cell size heterogeneity in steady-state populations. The results reveal the importance of mass resolution to correctly describe these mechanisms.

## Abstract (web summary)

A technological approach combines transport, guiding and focusing of individual bacteria from solution to ultrathin membrane resonators for dry mass determination of single cells with accuracy within 1% and throughput of 20 cells/min.

## Introduction

In spite of decades of research, how bacteria regulate the size remains an open question^1–5^. Most of our knowledge of cell growth and size regulation is an average description of cell populations. However, cell populations are intrinsically heterogeneous due to both stochastic and deterministic mechanisms. The resulting diversity plays an essential role in biological function. A major understanding on the mechanisms of bacteria size regulation requires of techniques that can precisely measure individual cells in populations^6^. Cell size can be quantified by either, volume or mass, however both parameters are extremely sensitive to the environmental conditions, such as humidity and osmotic pressure that modifies the water content that accounts for 60–80% of the total mass^7^. Dry mass, the mass of the non-aqueous content of the cell, has increasingly gained attention as it provides information on the amount of all constituents of bacterial cells, namely proteins, nucleic acids, carbohydrates and lipids^8^. The dry mass of a single cell can be regarded as the most precise indicator of the biosynthetic and degradative processes within a cell^6^. Several technologies have been recently developed for measuring the dry mass of single cells, such as suspended microchannel resonators, quantitative phase microscopy and mass spectrometry (MS) based techniques. The first approach determines the buoyant mass of single cells flowing through a suspended microchannel by measuring the variations in the resonance frequencies of the microchannel^9–15^. Determination of the dry mass of the bacteria requires measuring the same cell in two fluids of different density. This technique provides high mass resolution (<1 fg), however is technically complex. Quantitative phase imaging (QPI) uses light interferometry for measuring the phase shift of a light wave after passing through the cell^16–18^. The fact that the ratio between refractive index and cell biomolecular mass density falls within a very narrow range^19,20^, allows to compute the dry mass of the cells with high-throughput and high simplicity. However, QPI cannot be used for accurate mass determination of small cells such as bacteria due to the limited spatial resolution and phase noise. Finally, two basic approaches based on MS have been developed for measuring the individual mass of previously ionized biological particles in ultrahigh vacuum, one measures the mass-to-charge ratio of a single ion at several charge steps to infer the mass^21^, the other referred to as charged-detection (CD) MS simultaneously measure the charge and the mass-to-charge ratio of single ions^22–26^. The first method can obtain the mass of ions >100 MDa such as bacterial cells. However, this method has been little widespread due to its complexity, long measurement time per ion and the little number of measured ions, usually a few tens^25,26^. Conversely CD-MS has become the most widespread MS technique for measuring mass distributions of ions with masses well-above the MDa, such as large protein complexes and small viruses, due to the capability for measuring thousands of ions in periods of tens of minutes^22–25^. However, the mass resolution rapidly decreases for higher masses^25,26^, which prevents, with very few exceptions^27^, the measurement of large viruses with masses higher than 100 MDa (e.g. influenza virus, HIV, herpesviruses, SARS-CoV-2, Ebola-like viruses, etc.) and bacterial cells with masses from tens to hundreds of GDa.

Nanomechanical mass spectrometry (NMS) based on micro- nanoscale mechanical detectors has recently emerged as the only technique that can directly ‘weigh’ single biological particles from the nanometer scale to the micrometer scale, such as proteins, viruses and cells with unprecedented resolution and without any assumption on their physical properties^28–33^. In NMS, biological particles are gently ionized from aqueous solution into the gas phase by electrospray ionization (ESI) and subsequently guided to the surface of a micro- nanoscale mechanical resonator usually in (ultra)high vacuum, and very recently at atmospheric pressure^33^. As each particle accretes on the resonator, abrupt downshifts in the frequency of the vibration modes of the resonator are observed that are directly proportional to the mass of the particle irrespective of its charge state^28,34–36^. A major limitation of nanomechanical mass spectrometry is the poor detection efficiency defined as the fraction of the particles nebulized from the solvent that reaches the mechanical detector. Best reported detection efficiencies for biological particles are between 10^−9^ and 10^−7^, depending on the particle characteristics and experimental conditions, leading to both long analysis times and excessive sample consumption. The poor efficiency mainly stems from the compromise between the size of the mechanical resonator and the minimum detectable mass. The smaller the device, the more susceptible is the resonant frequency to minuscule added masses. However, the smaller the device the more unlikely that the particle accretes on the resonator. Strikingly, NMS has been based on 1D beam-like resonators with length-to-width ratio typically >10, a geometry that is clearly inefficient to achieve high capture area while preserving high mass sensitivity.

Here we propose the use of ultrathin (50 nm thick) 2D rectangular membranes (400 × 350 μm^2^), for nanomechanical mass spectrometry of bacteria. We find that these structures optimally satisfy the compromise between large capture area and high mass resolution. We develop an inverse problem theory to retrieve the individual mass of bacterial cells from the changes in the vibrational properties of this kind of structures. We apply our technique to two bacteria species, *Staphylococcus epidermidis* and *Escherichia coli K-12*. The results demonstrate the unparalleled potential of nanomechanical spectrometry based on ultrathin 2D resonators for rapid screening of the dry mass of microorganisms and for accurately describing the cell-to-cell variation.

## Results and discussion

### Instrument design

**Fig. 1:**
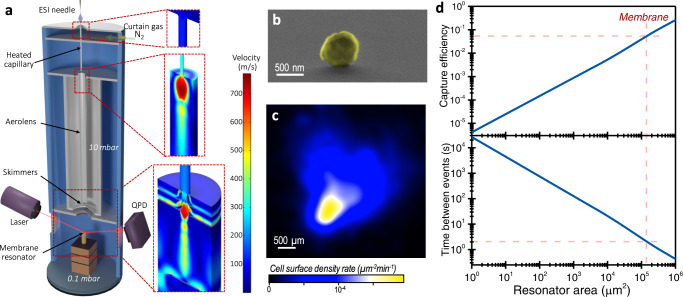
Design of the nanomechanical spectrometer for characterization of the dry mass of bacteria. **a** Schematics of the instrument (left) and estimation of the absolute flow speed calculated by FEM (right). The bacterial cells approximately follow the flow lines. **b** Scanning electron microscopy image of a *S. epidermidis* cell on a silicon surface at the detector position after nebulization. **c** Cell surface density at the detector position surface per unit time for a nebulization of *S. epidermidis*. The cell surface density is characterized by dark-field optical microscopy and subsequent digital counting of cell scattering spots. **d** Estimation of capture efficiency (top) and mean time between adsorption events (bottom) as a function of the detector area. The data are calculated by integrating the cell surface density rate (**c**) over a rectangle region that maximizes the number of collected cells. The dashed redlines mark the data for the membrane used in this work.

The instrument comprises four differential vacuum stages with decreasing pressure (Fig. 1a, left). The first stage houses an electrospray ionization (ESI) source at atmospheric pressure that generates mostly desolvated charged bacteria cells. The bacteria are then immediately attracted by the second stage, a capillary at 10 mbar that is heated at 150 °C to fully desolvate the microdroplets and to prevent sticking of bacteria to the internal wall of the capillary. The charged particles at the exit of the heated capillary come into the aerolens system comprising a long capillary followed by two consecutive skimmers^37^. Numerical simulations by the finite element method (FEM) show that the cells slow-down in the capillary from supersonic speeds (600–700 m/s) up to tens of m/s, achieving laminar flow (Fig. 1a, right). The skimmers give rise to a collimated narrow cell beam in the fourth differential vacuum chamber at 0.1 mbar that houses the resonator chip just 14 mm below. Near the resonator, bacterial cells are increasingly deaccelerated up to they ‘softly’ land on the resonator surface^38^. Soft-landing results into well-preserved cell morphologies with small contact areas with the resonator surface (Fig. 1b). In addition, the vacuum pressure in the detector chamber is more than four orders of magnitude higher than in any class of native mass spectrometry, low enough for removing the water content of the bacteria, while high enough for maintaining the native structure of the cells.

We characterized the detection efficiency of our instrument, which can be expressed as the product of the transmission efficiency and the capture efficiency. The first quantity is the fraction of the number of particles nebulized from the solvent to ambient conditions that reaches the detector vacuum stage. The transmission efficiency is independent of the mechanical detector, and it solely depends on the instrument architecture. The capture efficiency, in turn, is just determined by the ratio of the effective sensing area of the resonator to the cross-section of the charged cell beam at the resonator’s position. The cell distribution at the resonator position after nebulization was characterized by dark-field microscopy and image processing algorithms (Fig. 1c). The cells were located in a region with full width at half maximum (FWHM) of ~1.6 × 1.2 mm, as shown in Fig. 1c. for *S. epidermidis*. The data for *E. coli* follows a very similar behavior (Supplementary Fig. 1). We estimate a transmission efficiency of about 0.03% that is similar to previous state-of-the-art^29,30^. Most of the lost cells arise from the distance between the ESI needle and the heated capillary. The distance must be close enough to ensure high transmission, but also sufficiently large to avoid that water droplets enter in the vacuum system. The capture efficiency as a function of the detector area is estimated by integrating the cell surface density over a rectangular region that maximizes the number of collected cells (Fig. 1d, top graph). The capture efficiency is of about 5% for the ultrathin silicon nitride membranes used in our work, which is two orders of magnitude higher than in previous NMS works operating in vacuum^29,30^. We emphasize that the extraordinary large capture area does not compromise the mass resolution for characterizing bacteria due to the nanoscale thickness of the membranes and the narrow linewidth of the membrane vibration modes as shown below. We calculate the expected mean time between cell adsorption events as a function of the resonator area (Fig. 1d, bottom graph). In our experimental conditions (10^9^ cells/mL and flow rate of 300 nL/min), we predict a mean interval time between events of 2–3 s, which is similar to that experimentally observed. Beam-like resonators traditionally used for mass sensing, rarely exceeds capture areas of about 1000 µm^2^, giving mean interval time between events of minutes, and even hours for the smallest devices.

### Device design and operation

**Fig. 2:**
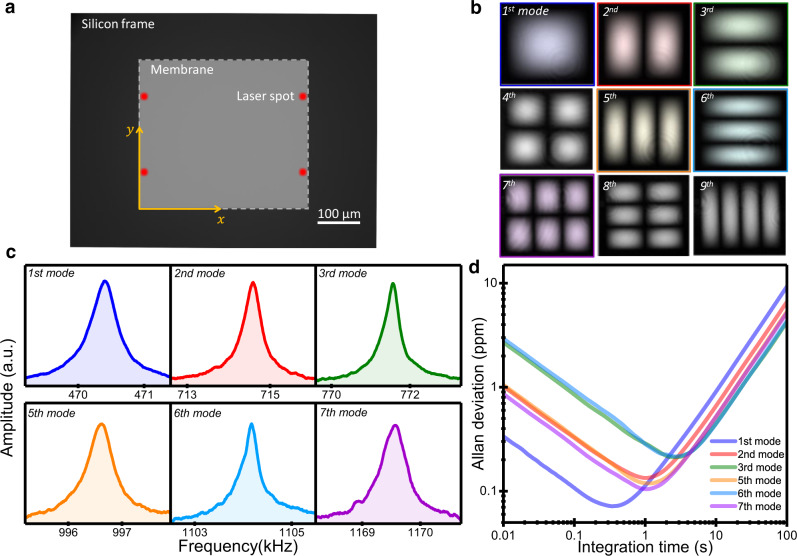
Characterization of ultrathin silicon nitride membrane resonators. **a** Optical microscopy image of the device. The silicon nitride membrane shows higher reflectivity than the silicon frame. The membrane is 50 nm thick and shaped as a rectangle with sides of 400 µm and 350 µm. The red circles mark the optimal position where the laser beam is focused to measure the six selected vibration modes (Supplementary Fig. 2). **b** The amplitude map of the first nine vibration modes of the membrane measured by stroboscopic digital holographic microscopy. **c** Frequency response of the selected vibration modes of the membrane. **d** Allan deviation of the selected vibration modes of the membrane.

We designed ultrathin 50 nm thick silicon nitride membranes with side lengths *L*_*x*_ = 400 *μm* and *L*_*y*_ = 350 μm (Fig. 2a). The small difference between the lengths, of 12.5%, plays a key role in the performance of our technology as discussed below. The vibration mode shapes of the membranes were experimentally measured by stroboscopic digital holographic microscopy (Fig. 2b)^39^. The experimental vibration mode shapes are well-described by linear elasticity theory of plates for the regime where the tension-induced stiffness of the membrane is much larger than the bending stiffness^40^. In this case, the eigenmodes are described by ${\psi }_{{ij}}=2{\sin }\left[\frac{i\pi }{{L}_{x}}x\right]{\sin }\left[\frac{j\pi }{{L}_{y}}y\right]$, where the mode identifier (*i*,*j*) refers to the number of antinodes in the *x* and *y* directions along the long and short sides of the membrane, respectively. The membrane displacement was measured by the laser-beam deflection method (Fig. 1a and Supplementary Fig. 2)^41^. In order to maximize the mass resolution by the inverse problem method, we simultaneously measured the frequency of six vibration modes: (1,1), (2,1), (1,2), (3,1), (1,3), and (3,2), by digital phase-locked loops (PLLs). The membrane oscillation was driven by a piezoelectric actuator just below the membrane chip. The fourth vibration mode, (2,2), was excluded because it could not be efficiently excited in our experimental conditions. The frequency spectra of the selected vibration modes are shown in Fig. 2c. The resonance frequencies are located at 470, 715, 770, 995, 1105 and 1170. These values approximately match the theoretical values given by ${\nu }_{{ij}}=\frac{1}{2}\sqrt{\left({\left(\frac{i}{{L}_{x}}\right)}^{2}+{\left(\frac{j}{{L}_{y}}\right)}^{2}\right)\frac{\sigma }{\rho }}$, for a tensile stress *σ* = 0.19 ± 0.02 GPa, assuming a density *ρ* = 2900 Kg m^−3^ (see Methods section). The corresponding Q-factors are very high in our low-vacuum conditions, of about 1500, 2700, 3000, 3000, 3500, and 4100, respectively. The frequency noise of the six vibration modes was estimated by calculating the Allan deviation^42,43^ (Fig. 2d). At short integration times, the Allan deviation decreases with the root square of the integration time and it is inversely proportional to the amplitude, which is consistent with frequency stability dominated by white phase noise. For integration times between 0.3 and 2 s, the Allan deviations reach the minimum of ∼10^−7^, and then they increase up to achieve an asymptotic regime where the Allan deviations linearly increase with the integration time due to drift-like sources of noise. The main source of drift arises from the effect of temperature on the stress of the membrane^44^. We minimize the eigenfrequency drift by waiting 3–4 h for thermal stabilization of the system before the measurements. The Allan deviation analysis provides a minimal detectable mass of ~0.7 fg for a particle located at the center of the membrane when measuring the fundamental resonance frequency.

We now turn our attention to the effect of the rectangularity of the membrane on the technique performance. In perfectly squared membranes, the vibration modes *i* ≠ *j*, are degenerate, i.e., modes (*i*,*j*) and (*j*,*i*) vibrate at the same frequency^45^. However, unavoidable small imperfections due to the fabrication process or small adsorbates on the membrane break the degeneration of these modes, splitting the degenerate resonance frequency in two very close resonance frequencies^46^. This configuration is highly unstable as any adsorption event can modify the frequency order of these two modes, leading to erroneous solutions by the inverse problem method. The rectangularity used here is high enough for stable breaking of the frequency degeneration, i.e., the vibration mode (*i*,*j*) has a lower frequency than the (*j*,*i*) for *i* > *j*, irrespective of the accumulated adsorption events. Very importantly, the rectangularity is sufficiently small to keep a quasi-symmetric capture area that is needed to optimize the detection efficiency. Moreover, the frequency density of vibration modes is significantly increased as each degenerate mode splits into two close, but uncoupled modes. Note that the frequency ratio between the 7th vibration mode and the fundamental mode is only of about 2.5, which significantly simplifies the frequency tracking of a high number of vibration modes as performed here.

### Instrument operation and data analysis

**Fig. 3:**
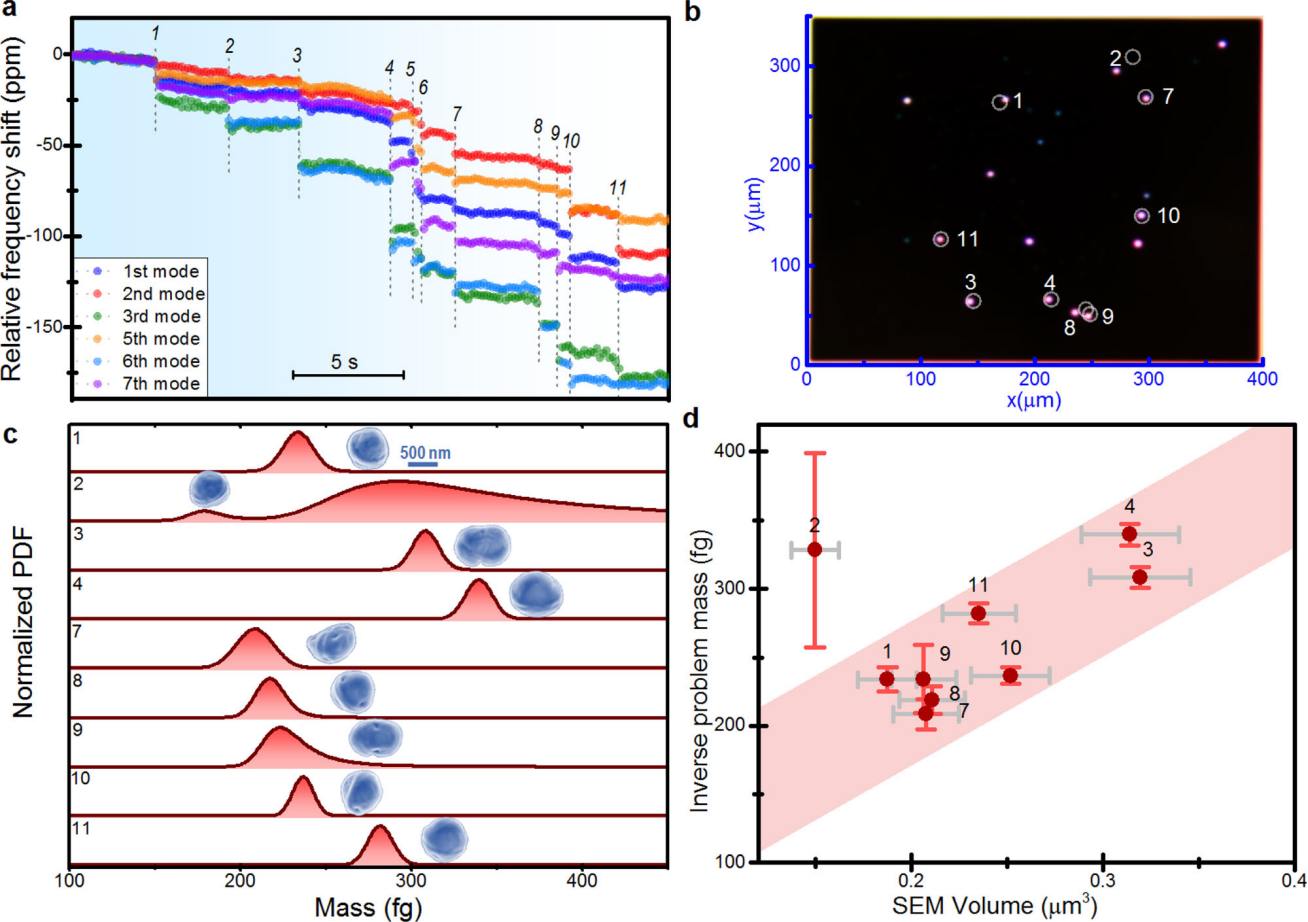
Validation of the inverse problem method. **a** Real-time record of the fractional frequency shifts of the selected six vibration modes of an ultrathin membrane during exposure to a flux of charged *S. epidermidis* cells. Soft-landing of individual cells on the membrane gives rise to quasi-instantaneous and simultaneous shifts of the tracked eigenfrequencies. The observed 11 tentative adsorption events are numbered. **b** Dark-field optical image of the membrane after the experiment shown in **a**. The positions of the bacteria estimated by the inverse problem method are shown as circles. Most of the predicted positions match dark-field spots that correspond with bacteria cells. Events 5 and 6 could not be resolved by the inverse method due to their short temporal separation. **c** Probability distribution function (PDF) of the individual bacteria mass obtained by the inverse problem method. The PDFs are normalized to the maximum. The insets show a detailed SEM image of the identified bacteria cells at each numbered position. **d** Mean mass of the bacteria derived by the inverse problem method vs the volume estimated from the SEM images of the bacteria. The mass error corresponds to the standard deviation of the dry mass PDFs. The volume is estimated by assuming an ellipsoid for the bacteria shape. We use the mean value of the in-plane diameters for the out-of-plane diameter. The volume error is obtained from the uncertainty in the three diameters of the ellipsoid.

We here describe the pipeline for determination of the dry mass of bacterial cells. The process starts by measuring the fractional frequency changes of the selected vibration modes of the membrane during nebulization of the bacteria solution. Figure 3a shows a short real-time record of these signals during nebulization of *S. epidermidis* cells. These curves are characterized by plateaus interspersed with quasi-instantaneous variations, referred here to as ‘frequency jumps’, that simultaneously occur in several vibration modes. The frequency jumps are identified as “tentative” cell adsorption events if the frequency change of at least three vibration modes is at least twice the standard deviation of their corresponding frequency fluctuations. In this experiment, 11 tentative adsorption events were identified in the 27 s time span. We chose a PLL integration time of 140 ms for measuring the resonance frequencies of the membrane, which provides a good compromise between accuracy and capability for resolving temporally close frequency jumps (see Fig. 2d). For highly tensioned membranes, particle adsorption induces changes in the kinetic energy due the added mass of the particle, whereas the potential energy is negligibly affected by the stiffness of the particle^34,35^. In these conditions, the relative frequency shift of a vibration mode induced by the adsorption of a particle at the membrane position (*x*_0_,*y*_0_) is given by,

1 $$\frac{\triangle {\nu }_{{ij}}}{{\nu }_{{ij}}}=-\frac{m}{2M}{{\psi }_{{ij}}\left({x}_{0},{y}_{0}\right)}^{2}$$

where *M* is the mass of the membrane and *m* is the mass of the particle. Notice that due to the inherent symmetry of the membrane, there exist four symmetric adsorption positions where the changes in the eigenfrequencies are identical. For the sake of simplicity, hereinafter the analysis is restricted to a quarter of the membrane. We briefly summarize here the inverse problem method, leaving the details for Supplementary Note 1. Resolution of the mass and position of the particle requires at least the measurement of three vibration modes. In the absence of noise, the inverse problem is *well posed*, i.e., the inverse solution (*m*,*x*_0_,*y*_0_) maps to a single forward solution $\left(\frac{\triangle {\nu }_{11}}{{\nu }_{11}},\frac{\triangle {\nu }_{21}}{{\nu }_{21}},\frac{\triangle {\nu }_{12}}{{\nu }_{12}}\right)$. Notice that this one-to-one bidirectional relation cannot be obtained with other choice of modes. The frequency noise makes the problem ill posed, the problem is not invertible for all possible frequency outputs, and the inverse solution is retrieved in terms of probability rather than a deterministic solution. The accuracy of the inverse problem method increases with the number of measured vibration modes as done here. Each frequency jump is characterized by a mean value and a standard deviation. The probability density function (PDF) of the fractional eigenfrequency shift vector $\hat{\Omega }=\left[\frac{\triangle {\nu }_{11}}{{\nu }_{11}},\frac{\triangle {\nu }_{21}}{{\nu }_{21}},\frac{\triangle {\nu }_{12}}{{\nu }_{12}},\frac{\triangle {\nu }_{31}}{{\nu }_{31}},\frac{\triangle {\nu }_{13}}{{\nu }_{13}},\frac{\triangle {\nu }_{32}}{{\nu }_{32}}\right]$, due to a particle adsorption is given by,

2 $${PDF}\left(\hat{\Omega }\right)=\frac{{{{{{{\rm{e}}}}}}}^{-\frac{\left(\hat{\Omega }-\hat{\triangle }\right){\Sigma }^{-1}{\left(\hat{\Omega }-\hat{\triangle }\right)}^{{{{{{\rm{T}}}}}}}}{2}}}{{\left(2{{{{{\rm{\pi }}}}}}\right)}^{\frac{{{{{{\rm{N}}}}}}}{2}}\sqrt{|\Sigma |}}$$

where $\hat{\triangle }$ is the vector of the mean frequency shift, Σ is the covariance matrix and *N* is the number of vibration modes. By substituting the fractional eigenfrequency vector, $\hat{\Omega }$, by its theoretical dependency explicited in Eq. (1), we build the joint probability density of the mass particle and adsorption position. Instead of selecting a recovery method such as the minimum-mean-squared error (MMSE), maximum likelihood (ML) or maximum a posteriori (MAP) probability^47^, we here separately provide the probability density functions for the mass and position of the bacterial cells.

The proposed inverse problem method is applied to the frequency jumps observed in Fig. 3a. Inverse solutions are found for all the events, except for the fifth and sixth that cannot be clearly resolved due to the short temporal separation, of about 400 ms. The adsorption positions with highest probability for the remaining events are compared to the positions of the scattering spots at the membrane obtained by dark-field microscopy after the experiments (Fig. 3b). We observe that the predicted positions are very close to darkfield hotspots. The corresponding mass probability distributions peak at masses between 200 and 350 fg (Fig. 3c). These peaks significantly broaden as the particles are near the membrane edges, where the amplitudes of the eigenmodes are smaller. This is notably the case of particle 2, whose derived position significantly deviates from its real position and its broad mass distribution prevents accurate quantification of its mass. Later, it is shown how poorly resolved events are excluded of the analysis based on mathematical criteria. We compare the masses obtained by the inverse method with the sizes obtained by SEM images of the identified bacteria on the membrane (insets of Fig. 3c). We find that *S. epidermidis* cells are present at different growth stages, consistent with steady-state population of asynchronously growing cells. For instance, cell 3 and cell 9 were under division. We find a good correlation between mean cell mass estimated by the inverse problem method and the sizes estimated from the SEM images. The slope between the predicted mass and the volume estimated from the SEM images is of about 800 ± 200 Kg/m^3^, similar to previously reported density values (Fig. 3d)^29,48^.

### Determination of the mass heterogeneity of bacteria populations

**Fig. 4:**
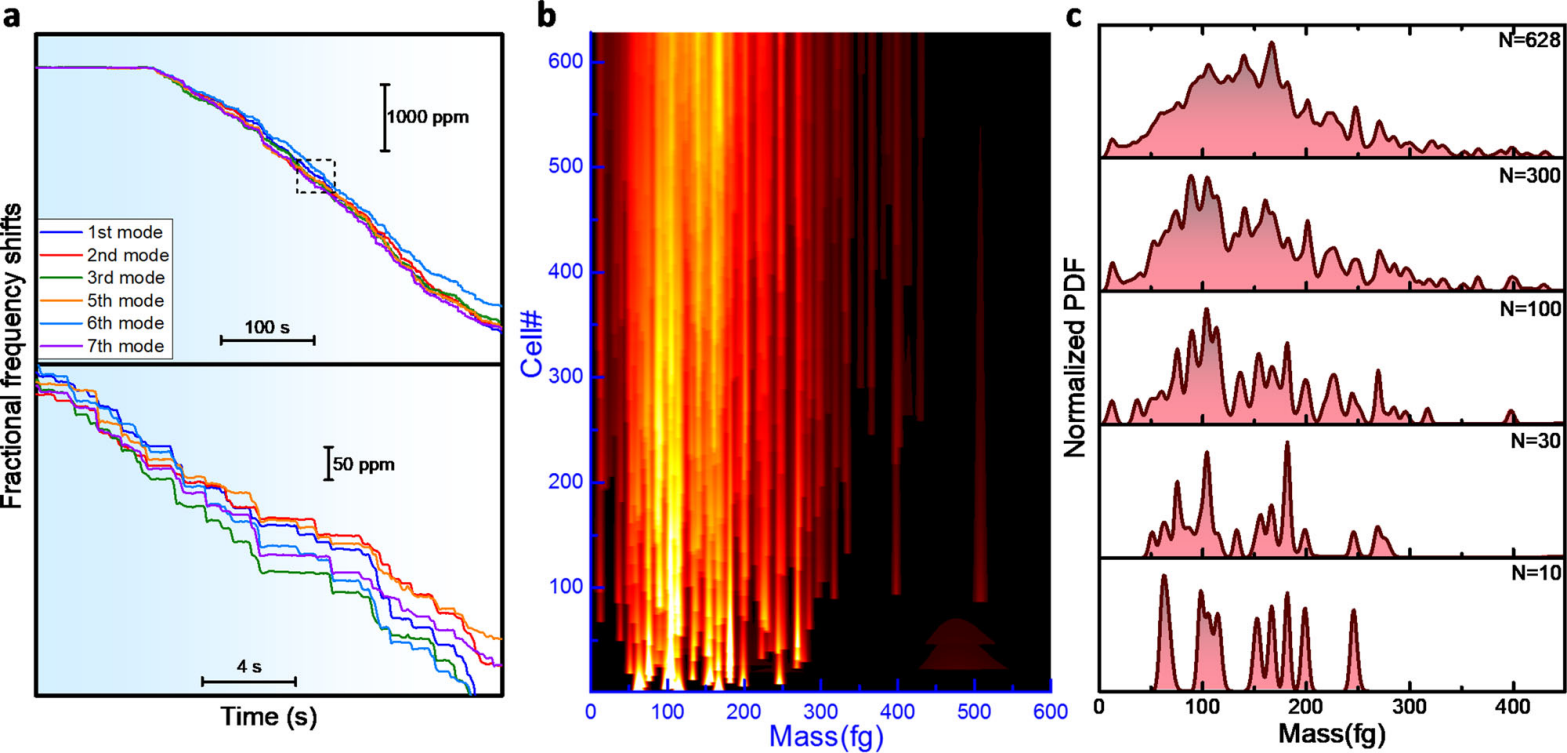
Nanomechanical spectrometry of a *S. epidermidis* population. **a** Real-time record of the fractional frequency shifts of the selected six vibration modes of an ultrathin membrane exposed to a flux of charged *S. epidermidis* cells. Abrupt downshifts of the eigenfrequencies are observed each few seconds. The dashed square of the top graph is zoomed in the below graph for easing the visualization of the adsorption events. **b** Sequential evolution of the probability distribution function (PDF) of the cell dry mass with the number of adsorbed cells (**c**). Normalized dry mass PDFs after adsorption of the first 10, 30, 100, 300 and 628 cells.

A 42 min real-time record of the fractional frequency changes of the selected vibration modes of the membrane during ESI of *S. epidermidis* is shown in Fig. 4a. About 900 tentative adsorption events were found. Not all events can be solved by the inverse problem method. A small portion are artifactual and may arise from spurious electronics’ noise, external mechanical disturbances or errors in the jump selection method. Other tentative adsorption events are true, but the frequency jumps are not precisely measured because the low signal-to noise ratio. This often occurs when the cells adsorb near the membrane edges, as shown in the previous section. We impose an admissibility condition to the tentative adsorption events based on two selection rules: i) the highest probability position of the particle obtained by the inverse problem method must be separated from the membrane edges more than 6% of the length and ii) the Pearson correlation coefficient between the eigenmode square amplitudes at the highest probability particle position and the experimental eigenfrequency downshifts must be higher than 0.9 (Supplementary Note 1). Assuming that the model that relates the particle parameters to the vibrational properties of the membrane is correct, a true adsorption event gives rise to a correlation coefficient of 1 for a noise-free measurement and it decreases with the frequency noise. Moreover, false events will result into anomalous low linear correlations and eventually in adsorption positions near the membrane edges. After imposing these selection rules, the number of valid events is reduced to 70% of the tentatively identified events. The resulting data shows that the single cell biomass is highly variable, leading to rapidly changing mass distributions with the number of measured cells for *N* ≲ 100 (Fig. 4b, c). The statistical parameters that describe the mass distribution start to reach the asymptotic regime for *N* ≳ 300. The final dry mass distribution (*N* = 628) exhibits a broad distribution with mean and standard deviation of 153 ± 69 fg, discretized with narrow peaks suggesting mass values with higher probability. This effect may be attributed to the high mass resolution, with a median of 3 fg, which enabled to resolve variations of 2% in the mass of single *S. epidermidis* cells. Indeed, the worst the mass resolution, the broader the probability distribution associated to a single cell, resulting into smoother mass distributions, such as those observed by optical and electric techniques^49,50^. Our mass accuracy is enough to describe the cell-to-cell variability, as shown below.

**Fig. 5:**
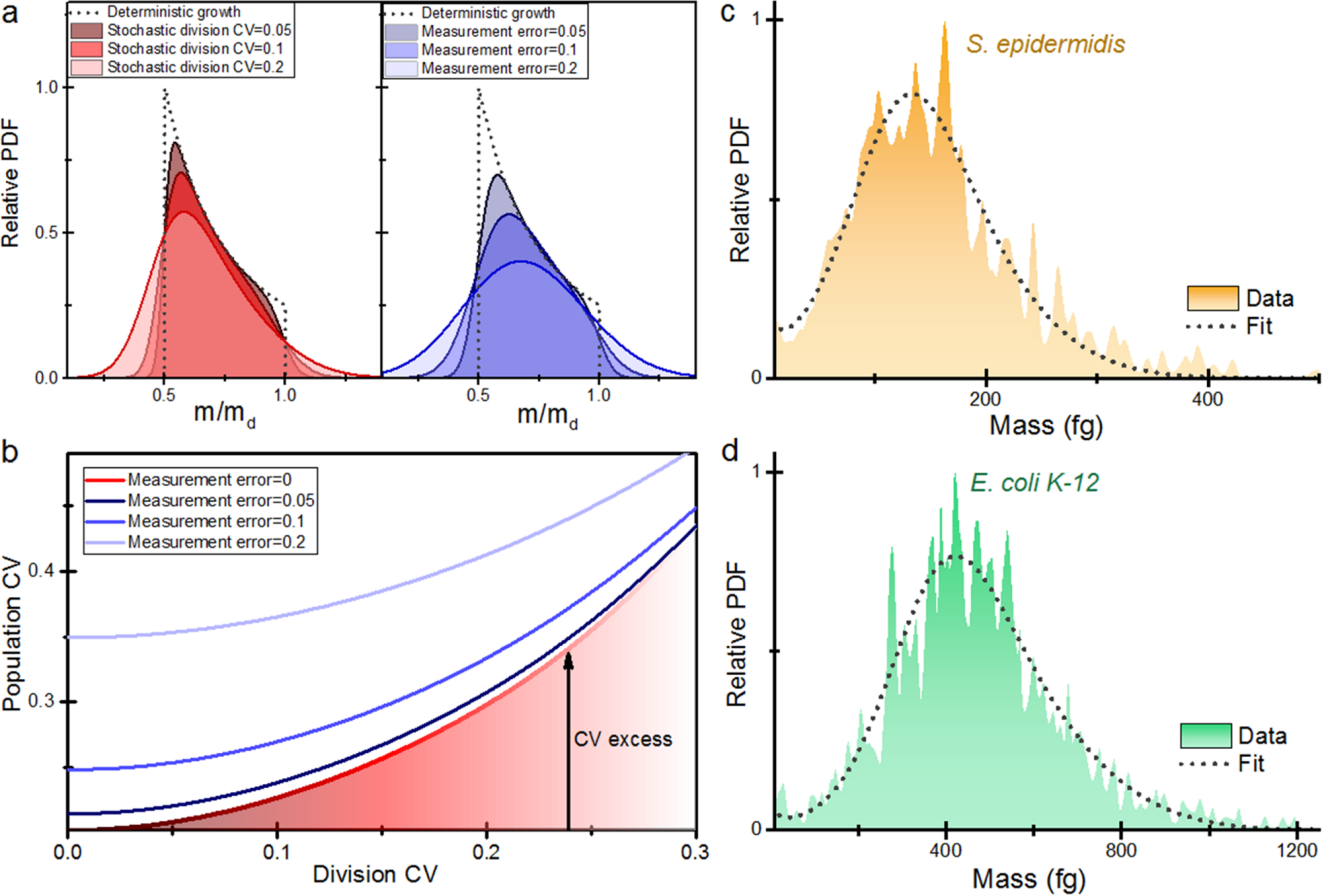
Cell dry mass stochasticity. **a** Left. PDF of the mass of a steady-state population of exponentially growing cells as a function of the variability of the cell mass at division as described by the Koch & Schaechter model. The dashed line represents the deterministic distribution. Right. Effect of the measurement noise on a deterministic distribution of cell masses. **b** Effect of cell division noise and measurement noise on the CV of the steady-state population of exponentially growing cells. **c**, **d** Relative PDF of steady-state populations of *Staphylococcus epidermidis* (*N* = 628) and *Escherichia coli K-12* (*N* = 685). The dashed line represents the fitting to Koch & Schaechter model.

We next investigated the nature of the dry mass heterogeneity of the bacteria cells. Cell growth and cell division are both subject to stochastic processes due to noise in the molecular circuit dynamics, partition noise and environmental fluctuations^1,2,49–51^. Cell size heterogeneity has been often quantified by statistical parameters, namely the coefficient of variation (CV). First attempts to relate the size distribution of steady-state populations of asynchronously growing cells to the source of stochasticity date back to the sixties in the works pioneered by Koch & Schaechter^52,53^. Unfortunately, these works were latter ignored, probably because the techniques at that moment, mainly the Coulter method, lacked of the needed accuracy. We here revisit these theoretical concepts for interpreting our data. Briefly, the steady-state mass distribution of exponentially growing cells that are free of stochasticity (deterministic or canonical distribution) is given by $\theta \left(m\right)=\frac{{m}_{d}}{{m}^{2}}$, when $\frac{{m}_{d}}{2}\le m\le {m}_{d}$ and zero otherwise, where *m*_*d*_ is the mass at division. Stochasticity in the cell growth and division makes that the mass distribution deviates from the deterministic distribution, and it is this deviation the key property for describing the intrinsic stochasticity of the cell population. Koch & Schaechter equation describes the effect of cell division stochasticity on the mass distribution (Fig. 5a, left),

3 $$\theta \left(m\right)=\frac{A}{{m}^{2}}{\int }_{m}^{2m}g\left(x\right){dx}$$

where *g*(*x*) is the mass probability density of the cells at the instant of division and *A* is a normalization constant. The beauty of this approximation is that the coefficient of variation (CV) for the deterministic growth is 0.2017. Therefore, intrinsic stochasticity is reflected into an excess of CV with respect to this value (Fig. 5b). We also examine the effect of mass resolution on the mass distribution (Fig. 5a, right) and on the CV (Fig. 5b). This effect has been overlooked in the past, but may lead to erroneous interpretation of the data. We find that mass resolutions above 5% can significantly distort the mass distributions and induce a false increase of the CV excess. This effect is not significant in our technique, but it may be important in other techniques with lower size resolution.

We apply the Koch & Schaechter model assuming a normal distribution of the division mass to the steady-state distributions of *S. epidermidis* (*N* = 628) and *E. coli K-12* (*N* = 685; Fig. 5c, d). Mean dry mass of *S. epidermidis* and *E. coli* were 153 and 469 fg, respectively. The coefficients of variation were 0.46 and 0.37, respectively. Although cell size distributions are usually fitted by normal and lognormal distributions, these statistical models are not substantiated in any growth model^50,51,53^. We find that Koch & Schaechter model fits with higher R-squared, and enables to infer information on the growth stochasticity parameters (Supplementary Fig. 3). The model provides a mean mass at the instant of division of 248 fg for *S. epidermidis* and 763 fg for *E. coli*. The CV of the division mass was 0.31 and 0.27, respectively. We remark again the relevance of measuring the dry mass with high accuracy to determine the sources of intrinsic stochasticity. Most studies on bacterial cell growth rely on real-time optical imaging of rod-like bacteria with lengths that range from 2 to 10 µm and widths of 0.2–1 µm. Cell volume is usually quantified by length assuming constant width due to the limited spatial resolution. Reported division length CV for rod-like bacteria lies between 10 and 15%^50,54^, from 2 to 3 times smaller than the CV of the division dry mass of *E. coli* reported here, which question the use of length as a reliable size indicator. Moreover, the limitation in spatial resolution of the used optical techniques prevents the study of cell growth of spherical bacteria smaller than 1 µm, such as *S. epidermidis* cells studied here.

## Conclusions

Both cell growth and cell division are subject to noise in the molecular circuitry as well as environmental fluctuations. However, cells are able to maintain their characteristic size. The mechanisms behind of cell size regulation are still poorly understood and remain as one of the big questions in biology. Despite the rapid advances in theory and technologies, the size of single bacterial cells cannot be rapidly measured with high precision. We show that size resolution plays a key role for correctly describing cell size variability. In fact, size resolution above 5% significantly modifies the statistical parameters of bacterial cell populations. Moreover, single cell mass is usually estimated by measuring its dimensions rather than measuring the mass of its biological constituents, i.e., the dry mass that is the most precise indicator. We introduce here a technological approach for characterizing the individual dry mass of hundreds of bacterial cells in few minutes with accuracy within 1%. These unique assets allow to scrutinize the mechanisms that allow bacteria cells to keep their size in their noisy context.

## Methods

### Design, fabrication, and characterization of the devices

The membrane geometry was designed in order to (i) maximize the capture area whilst maintaining enough mass resolution to accurately measure the dry mass of the bacteria cells, and (ii) to break the frequency degeneration in a stable way whilst maximizing capture area. These compromises were satisfied by ultrathin 50 nm thick silicon nitride membranes with length *L*_*x*_ = 400 μm and width *L*_*y*_ = 350 μm. The devices were custom-fabricated by Norcada Inc (Canada). The critical parameters of the device were characterized by Norcada Inc for the presented experiments. The final lengths were *L*_*x*_ = 403 ± 3 μm and *L*_*y*_ = 353 ± 3 μm. Membrane thickness measured by a spectroscopic ellipsometer was 53.7±2.0 nm. The silicon nitride stoichiometry was custom tuned by Norcada for having specific stress and density. The density was 2900±50 Kg/m^3^. The vibration modes of the membrane were characterized by reflection digital holographic microscopy (DHM R-1000, Lyncée Tec, Switzerland), with a 10x magnification objective lens (Leica Camera AG, Germany). The measurements were carried out in a home-made vacuum chamber at 10^−3^ torr. The pressure was kept by a dual-stage rotary vane vacuum pump (TRIVAC E2 D2,5E, Oerlikon Leybold Vacuum GmbH, Germany). The top of the vacuum chamber accommodates an antireflective-coated thin glass (ATG-401 from UQG Optics, United Kingdom) that enables DHM characterization. The membrane was driven by a piezoelectric actuator (miCos Iberia S.L., Spain), just below the device. The piezoelectric excitation of the membrane was synchronized with laser-pulsed stroboscopic illumination. The phase delay of eight oscillation cycles of the resonance modes were recorded and the vibration mode shape was computed (software developed by Lyncée Tec).

### Instrument

The nanomechanical spectrometer is composed of three basic components: the electrospray ionization (ESI) source, the vacuum chamber comprising three differential vacuum stages, and the detector consisting of an ultrathin 50 nm thick silicon nitride membrane and a laser-beam deflection system for measuring the vibrational properties of the membrane. The ESI source consists of a stainless steel metal needle (76 µm ID, 152 µm OD, 14.6 cm long, Thermo Fisher Scientific, 97,144–20,040) inserted into a stainless steel metallic tube (Merck, 56722) used to supply a sheath gas flow (dry N_2_). The ESI source is connected to a high pressure syringe pump (Base 120 Cetoni Gmbh, Germany) and to a high voltage power supply (HV-7010 Ioner, RAMEM). A fraction of the charged particles generated by ESI enter the vacuum chamber through the heated capillary (0.5 mm ID, 7 cm long) fabricated by Fasmatech (Greece). The ESI tip is separated by 10 mm from the heated capillary inlet. The bacterial cell solution was sprayed at a flow rate of 0.3 μL/min and applying a voltage of 2650 V between the ESI needle and the vacuum chamber entrance. The temperature of the heated capillary is set to 150 °C and the pressure is kept at 10 mbar by a rotary pump (D8T Trivac Oerlikon Leybold Vacuum Gmbh, Germany). The heated capillary is embedded in a coaxial tube used to flow a curtain gas (dry N_2_) towards the ESI source. The sheath and curtain gases were flown at 150 mL/min. The sheath gas assists in (i) directing the spray to the vacuum chamber and (ii) desolvating the charged droplets before entering into the vacuum chamber. The curtain gas, in addition to enhance the desolvation of the charged microdroplets, contributes to expel the entry of air contaminants into the heated capillary. The charged particles at the exit of the heated capillary come into the aerolens system (Fasmatech, Greece) consisting of a long capillary (3 mm ID, 24 cm long) designed for decelerate the charged particles and achieve the laminar flow regime, followed by two consecutive skimmer-shaped lens electrodes with inner diameters of 2 mm and 1.5 mm, respectively, designed for focusing the charged particles on the membrane detector placed 14 mm below in the detector vacuum chamber kept at 0.1 mbar by a turbomolecular pump (Turbovac 90i, Oerlikon Leybold Vacuum Gmbh, Germany), which is back-pumped by a mono stage oil-sealed rotatory pump (MS40+, Agilent technologies, Inc). A piezo-ceramic actuator (miCos Iberia S.L., Spain) is placed underneath the device to excite the vibration of the membrane. A 3D nanopositioner stage (ANC350 Piezo Controller, Attocube Systems AG, Germany) below the membrane/piezoelectric stack is used to align the resonator surface and the beam of charged bacterial cells.

The laser-beam deflection system comprises a power-variable 639 nm laser diode (Schäfter+ Kirchhoff Gmbh, Germany) placed outside of the vacuum chamber that is focused trough an optical window at the detector chamber into the membrane surface. The laser power was kept to 550 µW to avoid photothermal heating on the membrane that results into drift of the resonance frequencies. The spot diameter on the membrane was of about 4 µm and was focused on the regions marked in Fig. 2a (Supplementary Fig. 2). The reflected beam was externally collected through an opposite optical window at the vacuum chamber into an objective (10x, 0.28 NA, Mitutoyo, Japan) that collimates the laser beam into 1 mm diameter beam before hitting the center of a quadrant photodetector (S4349, Hamamatsu, Japan). The photocurrents generated on the upper and lower quadrants are separately amplified by two high-speed current-to-voltage amplifiers (DHPCA-100, FEMTO Messtechnik GmbH, Germany). The output voltage signals are connected to the differential input of a digital lock-in amplifier (H2FLI-PLL Zurich Instruments AG, Switzerland) that provides 2 phase-locked loops (PLLs) and 4 proportional-integral-derivative (PID) controllers. The first two resonance frequencies of the devices were tracked by the PLL systems and the other four were tracked by configuring the PID controllers to lock the phases.

### Sample preparation

Firstly, 10 mL of Luria-Bertani (LB) broth samples were inoculated with 100 µL of the stationary phase cultures of *E. coli K-12* and *S. epidermidis* strains. The prepared bacteria solutions were grown overnight at 37 °C under agitation. Then, *E. coli* solutions were centrifuged at 2500 rpm during 5 min at 25 °C and *S. epidermidis* solutions were centrifuged at 4400 rpm during 25 min at 25 °C. The supernatant was removed and the remaining solutions were resuspended in Milli-Q water. This process was repeated three times. Finally, the bacteria were resuspended in a solution 50% isopropyl alcohol/50% Milli-Q water, and the concentration was adjusted to 10^9^ cells per mL by measuring the optical density (OD) at 600 nm using a BioSpectrophotometer from Eppendorf (Germany).

### Statistics and reproducibility

The mass distributions shown in this work correspond to 628 *S. epidermidis* cells and 685 *E. coli cells*. Cell adsorption events were excluded from the analysis based on the mathematical criteria described in the main text and Supplementary Note 1. The shown data is consistent with previous repetition of the experiments (more than three for each bacteria).

### Reporting summary

Further information on research design is available in the Nature Research Reporting Summary linked to this article.

## Supplementary information

Supplementary Information

Description of Additional Supplementary Files

Supplementary Data 1

Reporting Summary

## Data Availability

The source data underlying Fig. 3d are shown in Supplementary Data 1. Any data generated or analyzed during this study that are not included in Supplementary Data 1 are available from the authors upon request.
