# Supplementary material for: High-throughput determination of dry mass of single bacterial cells by ultrathin membrane resonators
Source: Commun Biol. 2022 Nov 11;5:1227. doi: 10.1038/s42003-022-04147-5 (PMC9651879; doi:10.1038/s42003-022-04147-5)
Supplement: Supplementary file 1 — Supplementary Information [file 42003_2022_4147_MOESM1_ESM.pdf]

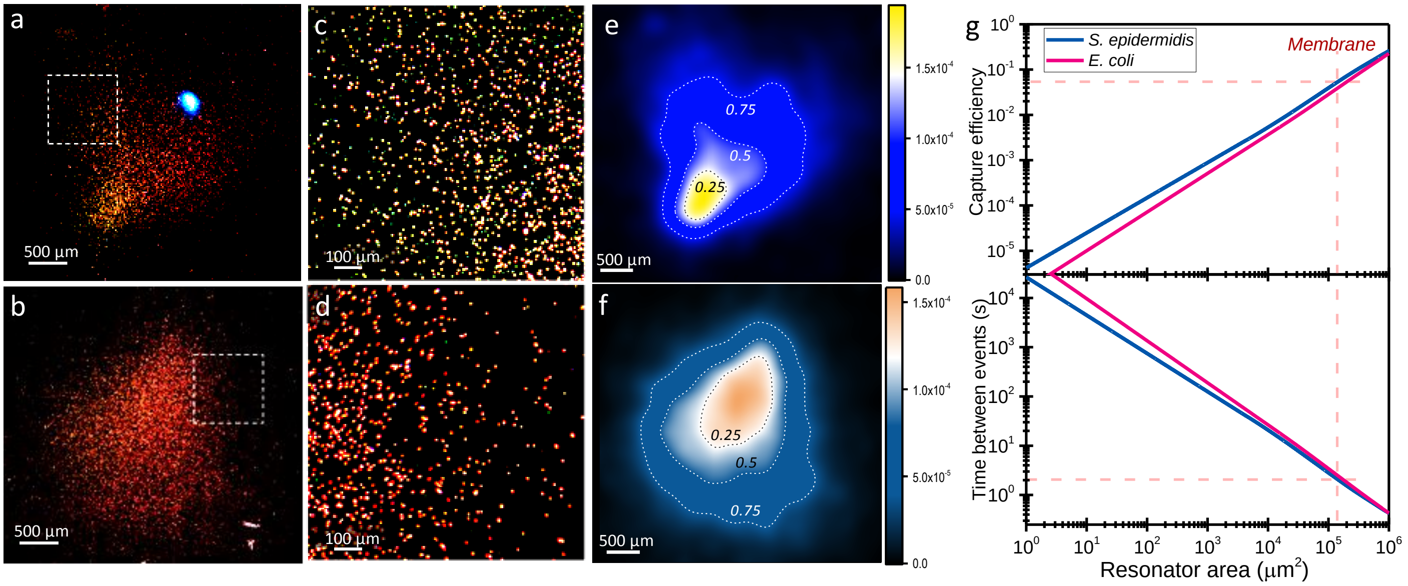

Supplementary Figure 1

Bacteria distribution on the sensor surface after ESI nebulization.

(a) & (b) Dark-field microscopy images of the surface after nebulization *Staphylococcus epidermidis* and *Escherichia coli K-12*, respectively. The used concentrations of bacteria were 4.4x 10^9 and 5x 10^9 per mL, respectively. The flow rate was 300 nL/min. (c) & (d) Zoomed images of the regions marked in (a) & (b), respectively. (e) & (f) Cell surface density per unit time of nebulization corresponding to images (a) & (b) obtained by an image processing algorithm written in Wolfram Mathematica language. Bacteria cells exhibit enhanced scattering that is reflected into image spots with a characteristic size and color intensity distributions. These spots are selected by the image processing algorithm to calculate the cell surface density. Isolines corresponding to 25%, 50% and 75% of the maximum are shown to ease the visualization of cell distribution. The full width at half maximum (FWHM) is of about 1.6 x 1.2 mm for *S. epidermidis* and 2.2 x 1.7 mm for *E. coli*. (g) Estimation of capture efficiency and mean time between adsorption events as a function of the detector area for *S. epidermidis* and *E. coli K-12* cells. The data are calculated by integrating the cell surface density rate, (e) & (f) over a rectangle region that maximizes the number of collected cells. The dashed redlines mark the data for the membrane used in this work.

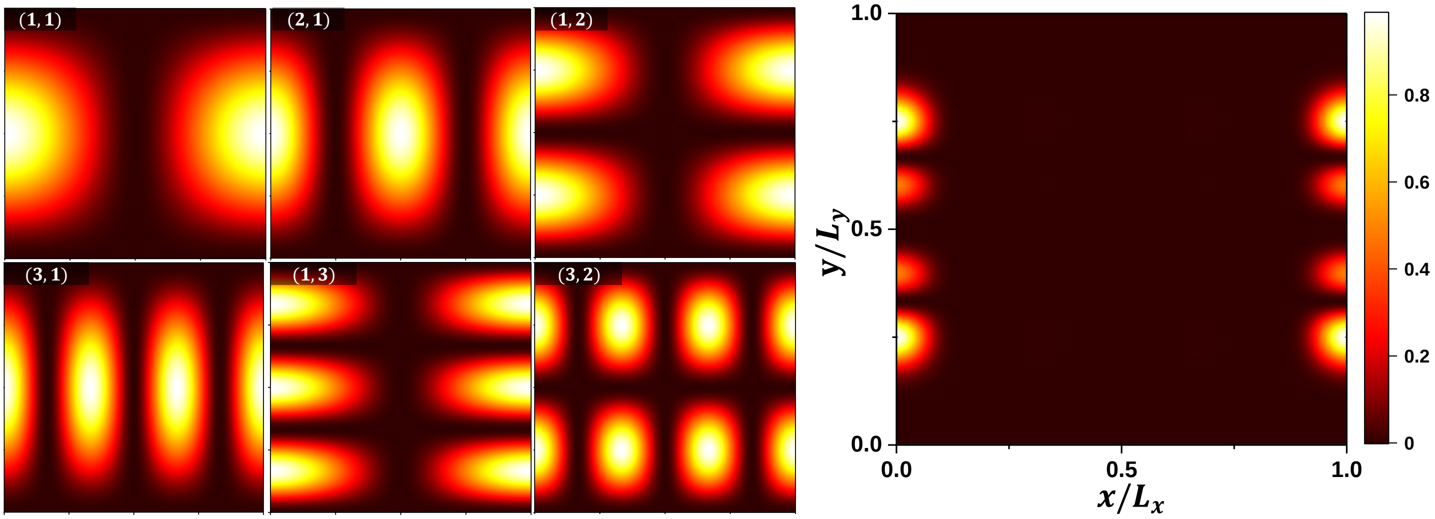

Supplementary Figure 2

Simultaneous detection of six vibration modes of the membrane by the laser-beam deflection method.

(Left) Relative square of the theoretical partial slope along x of the six selected vibration modes of the membrane. The Fourier components of the local x-slope associated to the selected vibration modes were measured by the laser-beam deflection technique and operating in parallel digital phase-locked loops (PLLs). In order to optimally measure the amplitude of these six vibration modes, the laser beam must be focused on membrane site that maximizes the product of Fourier components of the slope oscillation (Right). This condition is found by measuring near the x-boundaries ($x = 0$ or x= L_x) and $y/L_y \cong \frac{1}{2} \pm \frac{1}{4}$.

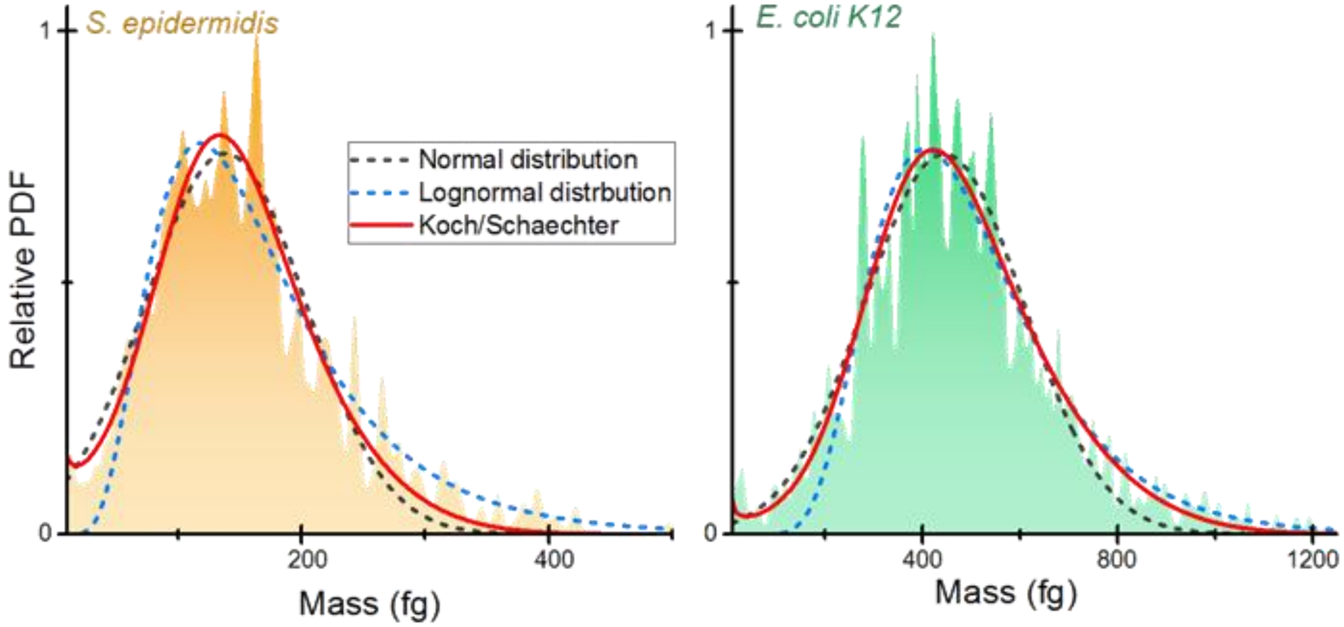

R-squared

Model	S. epidermidis	E. coli
Normal distribution	0.9584	0.9550
Log normal distribution	0.95743	0.9458
Koch/Schaechter model	0.9646	0.9582

Supplementary Figure 3

Fitting dry mass distribution of bacteria cells.

(Top) Relative probability density functions (PDFs) of steady-state populations of *Staphylococcus epidermidis* (N=628) and *Escherichia coli K-12* (N=685) (filled curves). The fitting functions corresponding to a normal distribution, lognormal distribution and the Koch & Schaechter model are plotted (see plot legend). (Bottom) Table showing the R-squared of the fittings. In general, the normal distribution fits better than the lognormal distribution. Koch & Schaechter model provides the best fitting, in particular for *S. epidermidis* cells.

Supplementary Note 1

Inverse problem method

The eigenmodes of a membrane are described by $\psi_{ij} = 2\, sin\left[\frac{i\,\pi}{L_x}x\right] sin\left[\frac{j\,\pi}{L_y}y\right]$, where the mode identifier (i, j) refers to the number of antinodes in the x and y directions along the length and width of the membrane, respectively. The adsorption of a particle at the membrane position (x_0, y_0) causes an abrupt change in the eigenfrequencies of the membrane. The relative frequency shift is given by,

$$\frac{\Delta\nu_{ij}}{\nu_{ij}} = -\frac{m}{2M}\psi_{ij}(x_0, y_0)^2 \qquad (1)$$

, where M is the mass of the membrane and m is the mass of the particle. Experimentally, the measured eigenfrequencies are subject to stochastic fluctuations of intrinsic and extrinsic nature. Therefore, we obtain a probability distribution of the relative frequency shifts instead of deterministic values. For each event, we build a Gaussian probability density function of the relative frequency shifts. The probability density function of the fractional eigenfrequency shift vector $\widehat{\Omega} = \left[\frac{\Delta\nu_{11}}{\nu_{11}}, \frac{\Delta\nu_{21}}{\nu_{21}}, \frac{\Delta\nu_{12}}{\nu_{12}}, \frac{\Delta\nu_{31}}{\nu_{31}}, \frac{\Delta\nu_{13}}{\nu_{13}}, \frac{\Delta\nu_{32}}{\nu_{32}}\right]$, due to the particle adsorption is given by,

$$PDF(\widehat{\Omega}) = \frac{e^{-\frac{(\widehat{\Omega}-\widehat{\Delta})\Sigma^{-1}(\widehat{\Omega}-\widehat{\Delta})^{\mathrm{T}}}{2}}}{(2\pi)^{\frac{N}{2}}\sqrt{|\Sigma|}} \qquad (2)$$

where $\widehat{\Delta}$ is the vector of the mean eigenfrequency shift, Σ is the covariance matrix and N is the number of vibration modes. By substituting equation (1) into (2), we obtain the probability density function of that the eigenfrequency vector change is caused by the adsorption of a particle of a mass m at position (x_0, y_0)

$$PDF(m, X_0, Y_0) = \frac{e^{-\frac{(\widehat{\Omega}(m,x_0,y_0)-\widehat{\Delta})\Sigma^{-1}(\widehat{\Omega}(m,x_0,y_0)-\widehat{\Delta})^{\mathrm{T}}}{2}}}{(2\pi)^{\frac{N}{2}}\sqrt{|\Sigma|}} \qquad (3)$$

We form probability distributions for the mass of the particle and the position by separately integrating over the whole respective domains,

$$PDF(m) = \int_0^{L_y}\int_0^{L_x} \frac{e^{-\frac{(\widehat{\Omega}(m,x_0,y_0)-\widehat{\Delta})\Sigma^{-1}(\widehat{\Omega}(m,x_0,y_0)-\widehat{\Delta})^{\mathrm{T}}}{2}}}{(2\pi)^{\frac{N}{2}}\sqrt{|\Sigma|}} dx_0 dy_0 \qquad (4)$$

$$PDF(x_0, y_0) = \int_0^{\infty} \frac{e^{-\frac{(\widehat{\Omega}(m,x_0,y_0)-\widehat{\Delta})\Sigma^{-1}(\widehat{\Omega}(m,x_0,y_0)-\widehat{\Delta})^{\mathrm{T}}}{2}}}{(2\pi)^{\frac{N}{2}}\sqrt{|\Sigma|}} dm_a \qquad (5)$$

In these experiments, we select events, in which the eigenfrequencies undergo an abrupt change above two times the standard deviation of the frequency in at least three modes. Some of these selected events are artifactual and are not caused by the adsorption of a particle. Other events correspond with the adsorption of a particle, but the inverse problem method gives a broad mass distribution due to several reasons such as an eventually increase of the frequency noise, the particle is adsorbed near the membrane edges where the changes of the eigenfrequencies are smaller or two particles are adsorbed with a temporal separation similar to the sampling time (140 ms), making difficult to separate the associated events. In order to discard both artifactual events and poorly resolved events, we establish a two mathematical criteria to select the valid and well-resolved events. Firstly, we discard those events, in which the position of the particle with highest probability obtained by the inverse problem method (x_m, y_m) (Eq. 4) is very near the membrane edges, in particular, those that give $x_m < 0.06L_x$, $x_m > 0.94L_x$, $y_m < 0.06L_y$or $y_m > 0.94L_y$. Second, the correlation between the theoretical changes of the eigenfrequencies (Eq. (1)) at the position of highest probability (Eq. (4)) and the experimental (mean) relative eigenfrequency shits must be sufficiently high. This criterion is mathematically written by calculating the Pearson correlation coefficient between the following vectors

$$\boldsymbol{u} = [0, \psi_{11}(x_m, y_m)^2, \psi_{21}(x_m, y_m)^2, \psi_{12}(x_m, y_m)^2, \psi_{31}(x_m, y_m)^2, \psi_{13}(x_m, y_m)^2, \psi_{32}(x_m, y_m)^2] \quad (5)$$

$$\boldsymbol{v} = [0, \Delta_{11}, \Delta_{21}, \Delta_{12}, \Delta_{31}, \Delta_{13}, \Delta_{32}] \quad (6)$$

The Pearson correlation coefficient must be higher than a threshold value, which satisfies a compromise between eliminating most of the artifactual and poorly resolved events and minimally perturbing the dry mass distribution of the cells. A very high value near 1 can eliminate true events that correspond to small values of the mass, shifting the mass distribution to larger masses. A too low value near zero implies a significant loss in mass resolution and accuracy. We find this compromise in our experiments by selecting those events that give a correlation coefficient higher than about 0.9.
