# Supplementary material for: High-throughput determination of dry mass of single bacterial cells by ultrathin membrane resonators
Source: Commun Biol. 2022 Nov 11;5:1227. doi: 10.1038/s42003-022-04147-5 (PMC9651879; doi:10.1038/s42003-022-04147-5)
Supplement: Supplementary file 2 — Description of Additional Supplementary Files [file 42003_2022_4147_MOESM2_ESM.pdf]

Description of Additional Supplementary Files

File name: Supplementary Data 1
Description: The source data underlying Figure 3d**.**
