# Supplementary material for: High-throughput determination of dry mass of single bacterial cells by ultrathin membrane resonators
Source: Commun Biol. 2022 Nov 11;5:1227. doi: 10.1038/s42003-022-04147-5 (PMC9651879; doi:10.1038/s42003-022-04147-5)
Supplement: Supplementary file 4 — Reporting Summary [file 42003_2022_4147_MOESM4_ESM.pdf]

nature portfolio

Corresponding author(s): Javier Tamayo

Last updated by author(s): Oct 12, 2022

Reporting Summary

Nature Portfolio wishes to improve the reproducibility of the work that we publish. This form provides structure for consistency and transparency in reporting. For further information on Nature Portfolio policies, see our Editorial Policies and the Editorial Policy Checklist.

Statistics

For all statistical analyses, confirm that the following items are present in the figure legend, table legend, main text, or Methods section.

n/a	Confirmed	
☐	☒	The exact sample size (*n*) for each experimental group/condition, given as a discrete number and unit of measurement
☐	☒	A statement on whether measurements were taken from distinct samples or whether the same sample was measured repeatedly
☐	☒	The statistical test(s) used AND whether they are one- or two-sided<br>*Only common tests should be described solely by name; describe more complex techniques in the Methods section.*
☒	☐	A description of all covariates tested
☒	☐	A description of any assumptions or corrections, such as tests of normality and adjustment for multiple comparisons
☐	☒	A full description of the statistical parameters including central tendency (e.g. means) or other basic estimates (e.g. regression coefficient) AND variation (e.g. standard deviation) or associated estimates of uncertainty (e.g. confidence intervals)
☒	☐	For null hypothesis testing, the test statistic (e.g. *F*, *t*, *r*) with confidence intervals, effect sizes, degrees of freedom and *P* value noted<br>*Give P values as exact values whenever suitable.*
☒	☐	For Bayesian analysis, information on the choice of priors and Markov chain Monte Carlo settings
☒	☐	For hierarchical and complex designs, identification of the appropriate level for tests and full reporting of outcomes
☒	☐	Estimates of effect sizes (e.g. Cohen's *d*, Pearson's *r*), indicating how they were calculated

Our web collection on statistics for biologists contains articles on many of the points above.

Software and code

Policy information about availability of computer code

Data collection	LabOne Software from Zurich InstrumTents , MEMS Analysis Tool from Lyncée Tec, NIS-Elements BR from Nikon, xT microscope Control from FEI
Data analysis	Wolfram Mathematica, COMSOL Multiphysics, OriginLab, Matlab, Python, MEMS Analysis Tool from Lyncée Tec

For manuscripts utilizing custom algorithms or software that are central to the research but not yet described in published literature, software must be made available to editors and reviewers. We strongly encourage code deposition in a community repository (e.g. GitHub). See the Nature Portfolio guidelines for submitting code & software for further information.

Data

Policy information about availability of data

All manuscripts must include a data availability statement. This statement should provide the following information, where applicable:

- Accession codes, unique identifiers, or web links for publicly available datasets
- A description of any restrictions on data availability
- For clinical datasets or third party data, please ensure that the statement adheres to our policy

The source data underlying Figure 3d are shown in Supplementary Data 1. Any data generated or analyzed during this study that are not included in Supplementary Data 1 are available from the authors upon reasonable request.

Human research participants

Policy information about studies involving human research participants and Sex and Gender in Research.

Reporting on sex and gender	N/A
Population characteristics	N/A
Recruitment	N/A
Ethics oversight	N/A

Note that full information on the approval of the study protocol must also be provided in the manuscript.

Field-specific reporting

Please select the one below that is the best fit for your research. If you are not sure, read the appropriate sections before making your selection.

☒ Life sciences ☐ Behavioural & social sciences ☐ Ecological, evolutionary & environmental sciences

For a reference copy of the document with all sections, see nature.com/documents/nr-reporting-summary-flat.pdf

Life sciences study design

All studies must disclose on these points even when the disclosure is negative.

Sample size	After data exclusion, 628 S. epidermidis cells and 685 E. coli cells were analyze to construct the mass distributions.
Data exclusions	Not all cell adsorption events can be solved by the inverse problem method. A small portion are artifactual and may arise from spurious electronics' noise, external mechanical disturbances or errors in the jump selection method. Other tentative adsorption events are true, but the frequency jumps are not precisely measured because the low signal-to noise ratio. This often occurs when the cells adsorb near the membrane edges, as shown in the previous section. We impose an admissibility condition to the tentative adsorption events based on two selection rules: i) the highest probability position of the particle obtained by the inverse problem method must be separated from the membrane edges more than 6% of the length and ii) the Pearson correlation coefficient between the eigenmode square amplitudes at the highest probability particle position and the experimental eigenfrequency downshifts must be higher than 0.9 (Supplementary Note 1).
Replication	To verify the reproducibility more than three of more experiments were previously performed and analyzed. Then, we optimized the instrument, methods and devices to perform the experiment shown in this work. We confirm that we can reproduce the data presented in the manuscript.
Randomization	Randomization was not required in our study
Blinding	Blinding was not required in our study

Reporting for specific materials, systems and methods

We require information from authors about some types of materials, experimental systems and methods used in many studies. Here, indicate whether each material, system or method listed is relevant to your study. If you are not sure if a list item applies to your research, read the appropriate section before selecting a response.

Materials & experimental systems

n/a	Involved in the study
☒	☐ Antibodies
☒	☐ Eukaryotic cell lines
☒	☐ Palaeontology and archaeology
☒	☐ Animals and other organisms
☒	☐ Clinical data
☒	☐ Dual use research of concern

Methods

n/a	Involved in the study
☒	☐ ChIP-seq
☒	☐ Flow cytometry
☒	☐ MRI-based neuroimaging
